# Predicting cysteine reactivity changes upon phosphorylation using XGBoost

**DOI:** 10.1002/2211-5463.13737

**Published:** 2023-11-20

**Authors:** Jing Cao, Yan Xu

**Affiliations:** ^1^ Department of Statistics University of Science and Technology Beijing China

**Keywords:** cysteine reactivity, machine learning, phosphorylation proteins, protein functions, XGBoost

## Abstract

Cysteine reactivity serves as a significant indicator of protein function and can be affected by phosphorylation events. Experimental approaches have been developed to investigate this effect, but the scale is still relatively limited. Machine‐learning approaches promise to accelerate the investigation of these phenomena. In this study, protein sequence information, distances to the closest phosphorylation sites, and the membership score of the intrinsically disordered region were used to represent the cysteine. Following the feature selection using an elastic net model, two groups of binary classifiers based on XGBoost were built to predict the occurrence and the direction of the reactivity change as a response to phosphorylation events, respectively. In addition, function enrichment analysis was performed on proteins/genes predicted to have reactivity changes. XGBoost performed the best in the independent test with AUC of 0.8192 and 0.9203 for the prediction of the change's occurrence and direction, respectively. The use of two binary classifiers successively resulted in an accuracy of 0.7568 in predicting whether reactivity would be unchanged, increased, or decreased. The enrichment analysis revealed the association of proteins carrying reactivity‐changed cysteine residues with various disease‐related pathways, particularly cancer, autosomal dominant diseases, and viral infections. Changes in cysteine reactivity influenced by phosphorylation are site‐specific and can be predicted by XGBoost algorithms. Our model provides an efficient alternative way to explore the cysteine reactivity upon phosphorylation at the proteome‐wide level, facilitating the investigation of protein functions and their clinical insights. Our code is available on GitHub (https://github.com/DarinaOsamu/predictors‐of‐cysteine‐reactivity‐changes).

## Graphical abstract

Cysteine reactivity is a significant indicator of protein function. We introduced the XGBoost algorithm to predict changes in cysteine reactivity influenced by phosphorylation events, which outperformed several other machine‐learning methods. A series of enrichment analyses were performed on cysteine predicted to have different changes, revealing the association of proteins carrying reactivity‐changed cysteine residues with various disease‐related pathways.

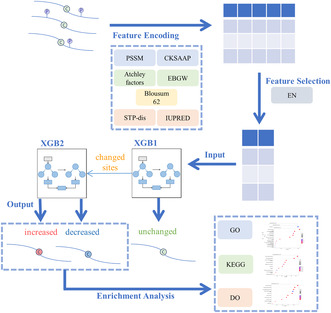

**Abbreviations**
ACC: accuracy
AUC: the area under the ROC curve
AUPR: the area under the PR curve
C, Cys: cysteine
DO: Disease Ontology
EN: elastic networks
GO: Gene Ontology
IUPRED: intrinsically unstructured proteins
KEGG: Kyoto Encyclopedia of Genes and Genomes
LR: logistic regression
MCC: Mathew's correlation coefficient
NB: Naive Bayes
PR: precision‐recall
PTM: post‐translational modifications
RF: random forest
ROC: the receiver operating characteristic
S, Ser: serine
SMOTE: synthetic minority over‐sampling technique
Sn: sensitivity
Sp: specificity
SVM: support vector machine
T, Thr: threonine
XGBoost: extreme gradient boosting

Cysteine (C) is a semi‐essential amino acid, which is a vital structural and functional portion of various peptides or proteins, and plays a significant role in the cellular redox balance [1]. Highly conserved cysteine residues are enriched at functionally important sites on proteins, whose reactivity can strongly indicate functionality [2].

The reactivity of cysteines can be regulated by several factors, such as Cu(II) and fumarate [1, 3]. Proteomic methods for targeting low‐abundance cysteine residues within their native environment and the effects of various physicochemical properties on cysteine reactivity have been explored at the subcellular level [2]. Molecularly, reactive oxygen and nitrogen species can dynamically regulate mitochondrial proteins via oxidative post‐translational modifications (PTMs) that occur on cysteine residues [4]. Phosphorylation is one of the most common and extensively studied PTMs *in vivo* primarily occurring on serine, threonine, and tyrosine residues. Recently, Kemper *et al*. [5] reported that changes in cysteine reactivity often occur during mitosis with a wide range of phosphorylation events, and further investigation has indicated the influence on cysteine of phosphorylation events happening in the same sequence. They characterized three groups of cysteine reactivity affected by phosphorylation [5]: increased and decreased cysteine reactivity, as well as other unchanged cysteines within the same protein. This study highlighted the association between phosphorylation‐dependent changes in cysteine reactivity and potential protein functions. This is the first study to measure the reactivities of cysteine upon phosphorylation event at large scale and provided valuable dataset for further investigation.

However, experimental methods for determining the reactivities of cysteine influenced by phosphorylation are still very challenging and time‐consuming. *In silico* computational methods such as machine‐learning approaches would provide the potential to accelerate the investigation. Currently, machine‐learning techniques are rarely used to predict the changes in cysteine reactivity under the influence of postmodifications, particularly of phosphorylation. Meanwhile, several machine‐learning algorithms have been developed to the prediction of cysteine reactivity. For example, Cy‐Preds [6] adopted the HAL‐cy method, which was mainly composed of two parts: the energy‐based part rooted in the evaluation of H‐bond network contributions and the knowledge‐based part rooted in similarity with known instances. They also developed new PSSM matrices for encoding protein sequences and establishing a set of rules for assessing cysteine reactivity based on the H‐bond network. sbPCR [7] combined basic local alignment search tools, truncated components of K‐space amino acid pair analysis, and the support vector machine to predict highly reactive cysteines. Moreover, two feature encodings were designed by Mapes Jr *et al*. [8]: the sequence‐based RAMseq and the structure‐based RAMmod. To assess reactivity, they considered oxidation to be a modification of cysteine and predicted whether cysteine was oxidized to evaluate the reactivity. In this way, they leveraged the PTM prediction methods to the prediction of cysteine reactivity, which is enlightening. All these approaches inspired us to develop a machine learning model designed specific to predict the phosphorylation‐dependent changes of cysteine reactivity.

Previous studies also provided useful information for protein sequences encoding, which could benefit to the development of the cysteine reactivities predictors. Besides the PSSM, K‐space amino acid pair and structure‐based RAMmod mentioned previous, Kemper *et al*. [5] revealed that cysteines with decreased reactivity are more likely than unchanged cysteines to reside in the disordered regions, whereas cysteines with increased reactivity are much less likely to be found in the disordered regions compared with unchanged cysteines. Meanwhile, cysteine reactivity is affected by the proximal S/T phosphorylation site [5].

In this study, we proposed to develop machine‐learning algorithms to predict phosphorylation‐dependent changes in cysteine reactivity by using protein sequences information only. We first constructed protein sequence features from three aspects: sequence characteristics, protein disordered regions, and distance to the nearest phosphorylation sites. Then, we built a predictor based on XGBoost after comparing several other computational predictors. Additionally, we applied our XGBoot model to predict the phosphorylation‐dependent changes in cysteine reactivity on the whole human phosphorylated proteome. The associated proteins with predicted cysteine reactivity changes were used to perform a functional enrichment analysis through Gene Ontology (GO), the Kyoto Encyclopedia of Genes and Genomes (KEGG) pathway, and Disease Ontology (DO). These phosphorylated proteins with changed reactivity enriched in cell division, signaling, and several disease‐related pathways, particularly cancer, autosomal dominant diseases, and viral infections. It reveals the clinical value of investigating phosphorylation on cysteine reactivity.

## Materials and methods

### Data collection and preprocessing

A total of 397 cysteine residues affected by phosphorylation events were obtained from Kemper *et al*. [5], including 89 increased sites and 308 decreased sites in 320 proteins. The protein sequences of these 320 proteins were received from the UniProt database [9]. After the full sequences was filtered out redundant sequences with a 30% threshold for pairwise sequence identity by cd‐hit [10], we got 80, 290, and 6357 cysteine residues with increased, decreased, and unchanged reactivity in 307 proteins, respectively. These proteins were truncated with a centered cysteine to a fragment length of 21 while missing amino acids were filled in with the pseudo amino acid “X.” Kemper *et al*. [5] suggested that whether the site is in a disordered region greatly influences the change in cysteine reactivity. Therefore, the prediction of intrinsically unstructured proteins (IUPRED) score of the target cysteine residues was adopted to evaluate the properties of the sites located within the disordered region from the IUPRED database [11].

Two binary predictors were designed: one to predict whether or not reactivity changes and another to predict the direction of these changes for affected residues. To ensure balanced samples, unchanged reactivity samples were randomly under‐sampled. Consequently, the benchmark dataset (Data S1) was composed of 370 unchanged sites and 370 changed sites (80 increased and 290 decreased reactivities).

We randomly selected 20% of the samples for each of the three categories of three types of samples, that is, increased, decreased, and unchanged. The selected samples were combined into an independent validation set. In this way, we obtained an independent validation set with 16 increased sites, 58 decreased sites, and 74 unchanged sites, and a training set with 64 increased sites, 232 decreased sites and 296 unchanged sites. The distribution of samples is the same for both sets.

Additionally, for the changed sites, a combined sampling method SMOTE‐Tomek [12] was adopted on the training set before training machine‐learning models. It is the combination of synthetic minority over‐sampling technique (SMOTE) and Tomek link.

### Feature construction

The proteins were encoded from three perspectives: sequence information, phosphorylation modification, and the location of the inherently disordered region.

1. Five encoding schemes including PSSM [13], CKSAAP [13, 14], Atchley factor coding [15], EBGW [16, 17], and Blousum62 [14, 17] were used to construct features from sequence characteristics, physicochemical properties, and evolutionary information [14, 17].
2. In the case of phosphorylation modification, with a focus on serine (S) and threonine (T) phosphorylation sites, the closest distance from the target cysteine residues to the phosphorylation S, T, and either of these two types of phosphorylation residues was obtained. When there are no S or T phosphorylation sites present on the sequences, the distance to the closest phosphorylation sites can be regarded as infinity. To avoid the incalculable condition caused by infinity, we applied reciprocals of the distance to its closest existing sites as a set of features named P‐dis, and the situation without a phosphorylation site was denoted by 0. The higher the value of P‐dis, the closer the target residue is to the phosphorylation site, and the maximum value is 1. The feature P‐dis has three‐dimensions, corresponding to the phosphorylation S, T, and either of them, respectively.
3. Previous study emphasized the importance of considering the location of inherent disorder regions. The IUPRED score represents the probability of the residue being in the disorder region, was thus adopted as one of our final feature [11].

We eventually obtained a feature set with 1468 dimensions by concatenating all the above features.

### Feature selection

High‐dimensional features could contain some redundant information, leading to a slow training speed and poor performance. In this paper, we used elastic networks (EN) to select features since they can automatically select variables while still maintaining model stability [18].

Two ENs were trained for the change's occurrence and their direction, respectively. Hyperparameters were determined by grid search based on 10‐fold cross‐validation. Then, the coefficient thresholds which could maximize EN scores were selected (Fig. S1). Only features with absolute coefficient values greater than the thresholds would be picked as the final feature sets for model training. Of the 1468 features, 22 were selected for occurrence prediction, and 167 for direction prediction.

### Classification engine

XGBoost [19] is a generalized boosting technique, which is an interactive ensemble model that focuses on the examples that the model fails to predict correctly [20]. The base learner of the algorithm is the decision tree and uses a forward stepwise algorithm and uses structural risk minimization to determine the parameters of the next decision tree $\Theta_{m}$, which is given by:

(1) $${\hat{\Theta }}_{m}={\arg\;\min}_{\Theta_{m}}\sum_{i=1}^{N}L\left(y_{i},{\hat{y}}_{i}\right)+\Omega \left(h_{m}\right),$$

where $L\left(y_{i},{\hat{y}}_{i}\right)$ is the loss function which measures the goodness of the prediction ${\hat{y}}_{i}$ and the object $y_{i}$, $\Omega \left(h_{m}\right)$ is a regularization term of the m‐th decision tree $h_{m}$ [19]. The regularization term is chosen as:

(2) $$\Omega \left(h_{m}\right)=\gamma T+\frac{\lambda }{2}\sum_{j=1}^{T}\omega_{j}^{2},$$

where T is the number of leaf nodes and $\omega_{j}$ is the output value of the j‐th leaf node, γ and λ are regularization parameters that must be chosen appropriately [21]. For training, we use the default hyperparameters in xgboost (version 1.6.0) in python, except for the “n_estimators” is determined by 10‐fold cross‐validation, while “max_depth” and “min_child_weight” hyperparameters, which are determined using a grid search.

**Fig. 1:**
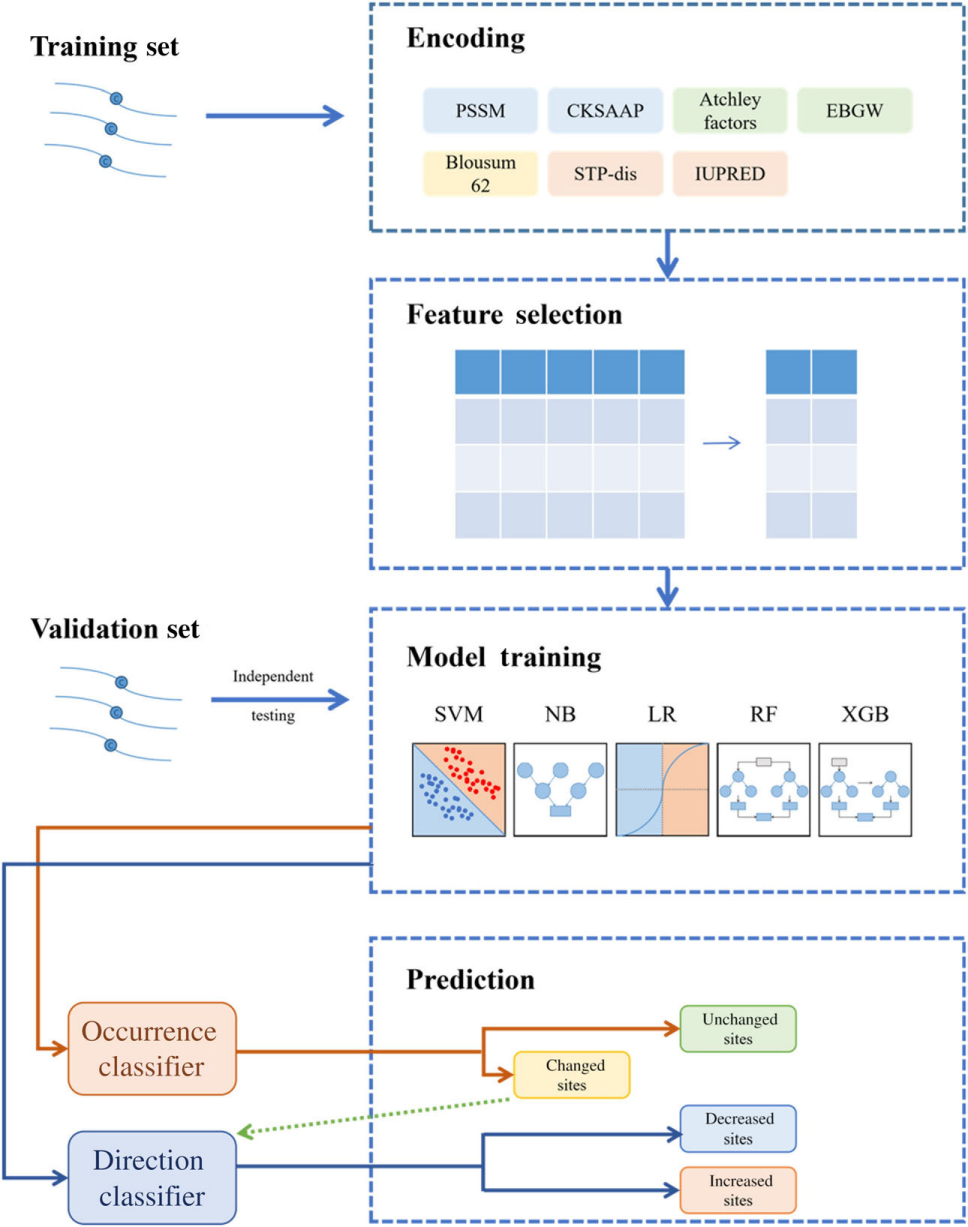
Schematic representation of the proposed method.

To predict the phosphorylation‐dependent changes in cysteine reactivity, we adopted the XGBoost to predict the occurrence and the direction of reactivity changes respectively, which could be synthesized to obtain the results of tri‐classification. On the contrary, we also added several machine‐learning methods to train the model. The model structure is shown in Fig. 1.

### Prediction metrics

In the statistical performance of binary classification, sensitivity (Sn), specificity (Sp), accuracy (ACC), and Mathew's correlation coefficient (MCC) are typically used as assessment metrics, which are defined as follows:

(3) $$\mathrm{Sn}=\frac{\mathrm{TP}}{\mathrm{TP}+\mathrm{FN}},$$

(4) $$\mathrm{Sp}=\frac{\mathrm{TN}}{\mathrm{FP}+\mathrm{TN}},$$

(5) $$\mathrm{ACC}=\frac{\mathrm{TP}+\mathrm{TN}}{\mathrm{TP}+\mathrm{TN}+\mathrm{FP}+\mathrm{FN}},$$

(6) $$\mathrm{MCC}=\frac{\mathrm{TP}\times \mathrm{TN}-\mathrm{FP}\times \mathrm{FN}}{\sqrt{\left(\mathrm{TP}+\mathrm{FP}\right)\left(\mathrm{TP}+\mathrm{FN}\right)\left(\mathrm{TN}+\mathrm{FP}\right)\left(\mathrm{TN}+\mathrm{FN}\right)}},$$

where TP, TN, FP, and FN, respectively, denote the number of true positives, true negatives, false positives, and false negatives. We also used the receiver operating characteristic (ROC) curve as well as the area under the ROC curve (AUC) [14, 22] to measure of the quality of the predicted models. The precision–recall (PR) curve and the area under the PR curve (AUPR) can also be applied for evaluation.

Accuracy and the precision, recall, and F1‐score of each category were used to evaluate the performance of the tri‐classifier, which are defined as follows:

(7) $$\text{Accuracy}=\frac{{\mathrm{TP}}_{-1}+{\mathrm{TP}}_{0}+{\mathrm{TP}}_{1}}{{\mathrm{TP}}_{-1}+{\mathrm{FP}}_{-1}+{\mathrm{TP}}_{0}+{\mathrm{FP}}_{0}+{\mathrm{TP}}_{1}+{\mathrm{FP}}_{1}},$$

(8) $${\text{Precision}}_{i}=\frac{{\mathrm{TP}}_{i}}{{\mathrm{TP}}_{i}+{\mathrm{FP}}_{i}},$$

(9) $${\text{Recall}}_{i}=\frac{{\mathrm{TP}}_{i}}{{\mathrm{TP}}_{i}+{\mathrm{FN}}_{i}},$$

(10) $$F1‐{\text{score}}_{i}=\frac{2\times {\text{Precision}}_{i}\times {\text{Recall}}_{i}}{{\text{Precision}}_{i}+{\text{Recall}}_{i}},$$

where the subscript i=−1,0,1, respectively, denote the category of decreased, unchanged, and increased. For example, ${\mathrm{TP}}_{-1}$ denotes the number of true positives of the decreased category.

## Results and Discussion

**Fig. 2:**
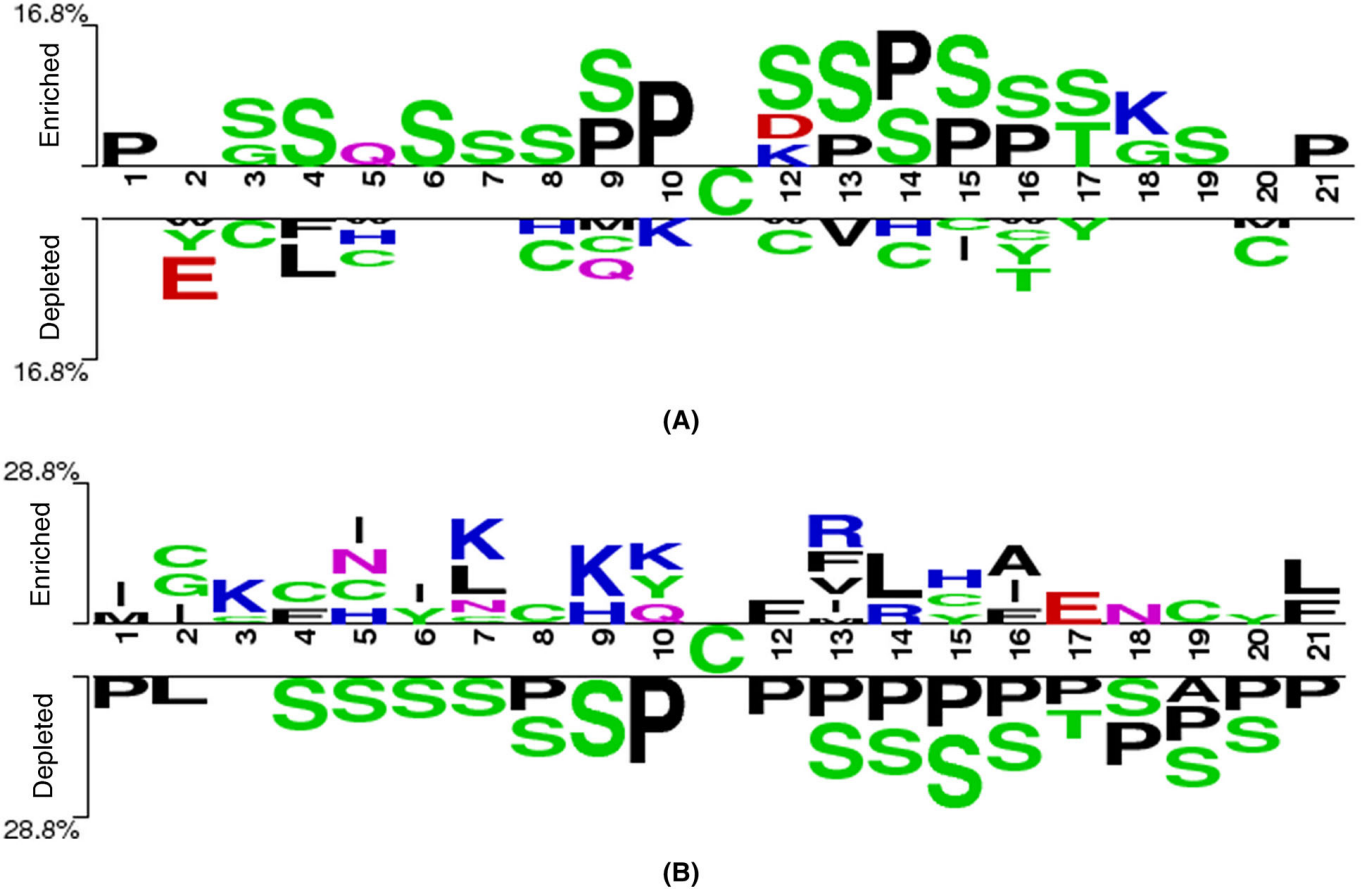
Sequence logo illustration generated by Two Sample Logos. (A) Samples with changed reactivity are positive, and the samples with unchanged reactivity are negative. (B) Samples with increased reactivity are positive, and samples with decreased reactivity are negative.

We first checked the sequence preference of the cysteines with reactivity changes upon phosphorylation. The sequence‐specific logos of different groups of cysteines were generated using the amino acid sequences consisting of 10 residues upstream and downstream to the cysteines by Two Sample Logo accordingly (Fig. 2). When comparing the cysteine with changed reactivities and unchanged reactivities, a distinct preference for prolines and serines is observed for the changed cysteines while random amino acid distributions are displayed for the unchanged cysteines. Particularly, serines are enriched in most of the neighboring positions of cysteines while prolines show enrichments in the closest positions to cysteines. Moreover, when comparing the decreased and increased reactivities changes of cysteines, prolines, and serines are enriched mainly for the decreased changed cysteines. Prolines are predominantly observed downstream of the cysteines, whereas serines are distributed in both upstream and downstream positions relative to the serines. Overall, the results show that the neighborhoods of sites with decreased reactivity exhibit a strong preference for P and S.

We then performed a feature selection using elastic net. The absolute value of each feature coefficient presents its importance in the prediction. The 10 most important features in descending order of their absolute values are shown in Table S1. The form “aa1aa2” indicates amino acid pairs spaced at 0 and the form “aa1_aa2” indicates amino acid pairs spaced at 1. It demonstrates the protein's disordered region's location, the distance to the phosphorylation site, and the amino acid pair composition are the three most important features for cysteine reactivity prediction.

We also explored the distribution of the above features among different classes. First, in both classification tasks, the IUPRED score show the greatest significance (Fig. S2). It validates that cysteine sites with decreased reactivity are more likely to be found in the disordered region, in agreement with previous research reports [5]. Furthermore, both the characteristics S‐P_dis and S/T‐P_dis are important for predicting whether reactivity is changed. As shown in Fig. S3, the P_dis values of samples with unchanged reactivity are more concentrated around 0, indicating that sites with unchanged reactivity are usually distant from S and T phosphorylation sites. This observation verifies the effect of the nearest phosphorylation site on cysteine reactivity.

To show the differences in amino acid pair composition with changed and unchanged reactivity more clearly, we plotted the differences in mean values for the most important amino acid pairs (Fig. S4a). In the same way, we plotted the differences in the mean values of the 9 most important amino acid pair compositions between the sample sets with increased and decreased reactivity (Fig. S4b). It can be seen that the amino acid pairs “SS,” “TS,” and “VC” distribute more in the reactive changed samples, while the amino acid pairs “NK,” “A_Y,” “L_C,” and “E_L” are less. The samples with increased reactivity tend to more “IY,” “A_D,” “IK,” “P_S,” “P_S,” “H_M,” “QD,” “TI,” and “DQ,” while “SS” is more abundant in the decreased reactivity.

To evaluate the performance of XGBoost, we also trained the binary classifiers of the occurrence and the direction of the change based on support vector machine (SVM) [23], Gaussian Naive Bayes (NB) [24], Logistic Regression (LR) [25], and random forest (RF) [26] to compare.

1. Results on change occurrence prediction

**Fig. 3:**
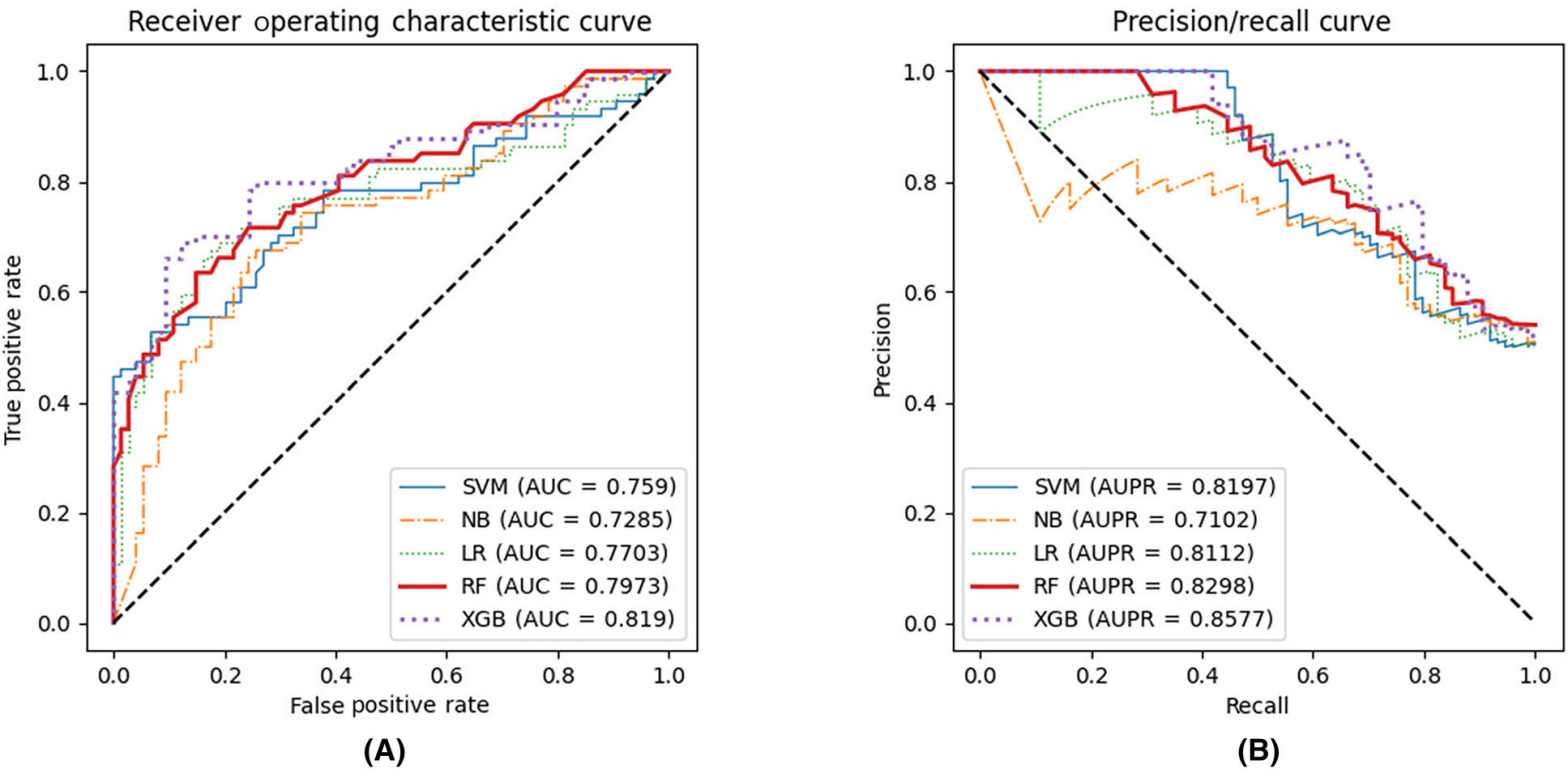
The performance of the occurrence classifiers after feature selection. (A) ROC curve of the occurrence classifiers after feature selection. (B) PR curve of the occurrence classifiers after feature selection.

We first compared the performance of models on predicting the occurrences of the reactivity change. Although the AUC of XGBoost algorithm decreased slightly from 0.8218 to 0.8190 after removing the redundant features, it maintained the superiority over other algorithms (Fig. 3), while the accuracy of XGBoost improved from 0.7365 to 0.7703, and the sensitivity improved from 0.7027 to 0.7838. Detailed values are shown in Tables S2 and S3. We chose the XGBoost algorithm as the final model for predicting the occurrence of changes of reactivities.

2 Results on prediction of the directions of changes

**Fig. 4:**
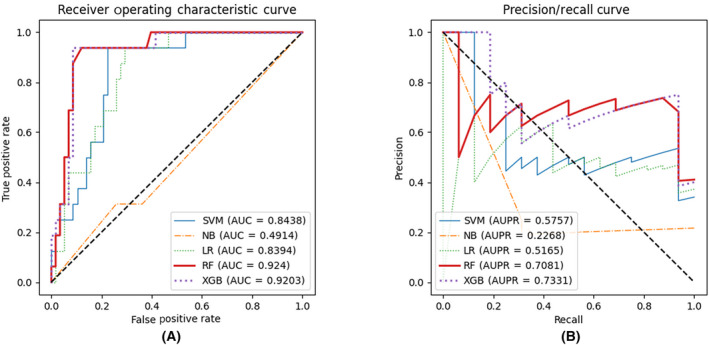
The performance of direction classifiers after SMOTE‐Tomek resampling and feature selection. (A) ROC curve of direction classifiers after resampling and feature selection. (B) PR curve of direction classifiers after resampling and feature selection.

We then compared the performance of different models on the task of predicting the direction of reactivity changes. The performance of the models after SMOTE‐Tomek and feature selection on the independent validation set were shown Fig. 4, while detailed ROC AUC and PR AUC values were shown in Table S6. The sensitivity and specificity of the XGBoost exceeded 0.9. The Random Forest and XGBoost perform best with regarding to the AUC score, and the Random Forest algorithm has a slightly better AUC value. With the same specificity of 0.9138, Random Forest achieves only a sensitivity of 0.6875, which is much lower than that of XGBoost at 0.9375.

3 Results on hierarchical tri‐classification

First, we use the occurrence classifier to predict whether the reactivity of cysteine changed. Cysteine sites that predicted to undergo changes are subsequently put into the direction classifier to obtain the final hierarchical tri‐classification results. It achieves 0.7568 accuracy for the tri‐classification on the independent validation set (Table 1).

**Table 1:** Results of the concatenated classifier of XGBoost.

Category	ACC	Precision	Recall	F1‐score
Decreased	0.7568	0.7705	0.8103	0.7899
Unchanged		0.7778	0.7568	0.7671
Increased		0.6000	0.5625	0.5806

In our model based on XGBoost, the AUC reaches 0.8192 and 0.9203 on the occurrence and direction predictions, respectively, and the tri‐classification reaches 0.7568 on the accuracy. A detailed comparison without treatment of dataset imbalance or feature selection is provided in Tables S3–S7. After removing the redundant features, the occurrence classification performances of Naive Bayes and Logistic Regression improve significantly (Table S3). The indexes of XGBoost also improve significantly, although the specificity and AUC decrease slightly. The performance metrics of each direction predictor trained without SMOTE‐Tomek resampling nor feature selection evaluated on the independent validation set are shown in Table S4. XGBoost shows a clear advantage on this classification task. Resampling improves the performance of Logistic Regression and Random Forest significantly, but only slightly for XGBoost in terms of AUC and AUPR (Table S5). Nevertheless, considering all metrics together, XGBoost still show the best performance, especially when several other algorithms has low sensitivity.

XGBoost has excellent performance, but SMOTE‐Tomek resampling seems to just provide a slight improvement. However, the elastic network used for feature selection has also been resampled. To compare the model without resampling at all and our model with resampling, we added the XGBoost classifier without resampling and with features selected by the elastic network also without resampling. A total of 136 features were selected from 1468 for model construction. After comprehensive consideration of all indicators, the XGBoost classifier with resampling and feature selection is still more effective (Table S7).

Different from the direct tri‐classification method, our hierarchical tri‐classification method carries out data preprocessing and feature selection for two different classification levels, respectively. To prove the superiority of our classifier to the model that directly carries out tri‐classification, we constructed models of direct tri‐classification based on the XGBoost in three conditions, respectively (Table S8). Due to the large gap between the minimum sample number and the other two categories, the performance of the classifier is adversely affected. The accuracy and recall of the samples with increased reactivity are very low, and the performance improvement is very limited even with resampling. We also noticed that the two binary classifiers performed obvious promotion after feature selection, while the direct tri‐classifier's performance was comprehensive down. It might be that the occurrence and the direction of cysteine reactive changes focus on different characteristics, and direct tri‐classification cannot select features according to the specific problem, so features with critical information are not successfully screened out. The concatenated classifier of XGB has higher accuracy than the direct classifier, and can better predict reactive increased samples with similar performances of the prediction of decreased and unchanged samples.

The effect of different fragment lengths on the performance of the classifier was also tested (Fig. S5, Tables S9–S11). With lengths of 21 and 23, the classifier achieves the highest AUC and AUPR for the occurrence and the direction of changes, respectively. The concatenated classifier with a length of 21 performs best according to ACC and F1‐score. Overall consideration, the peptide of length 21 is most suitable for our classifier.

### Enrichment analysis

**Fig. 5:**
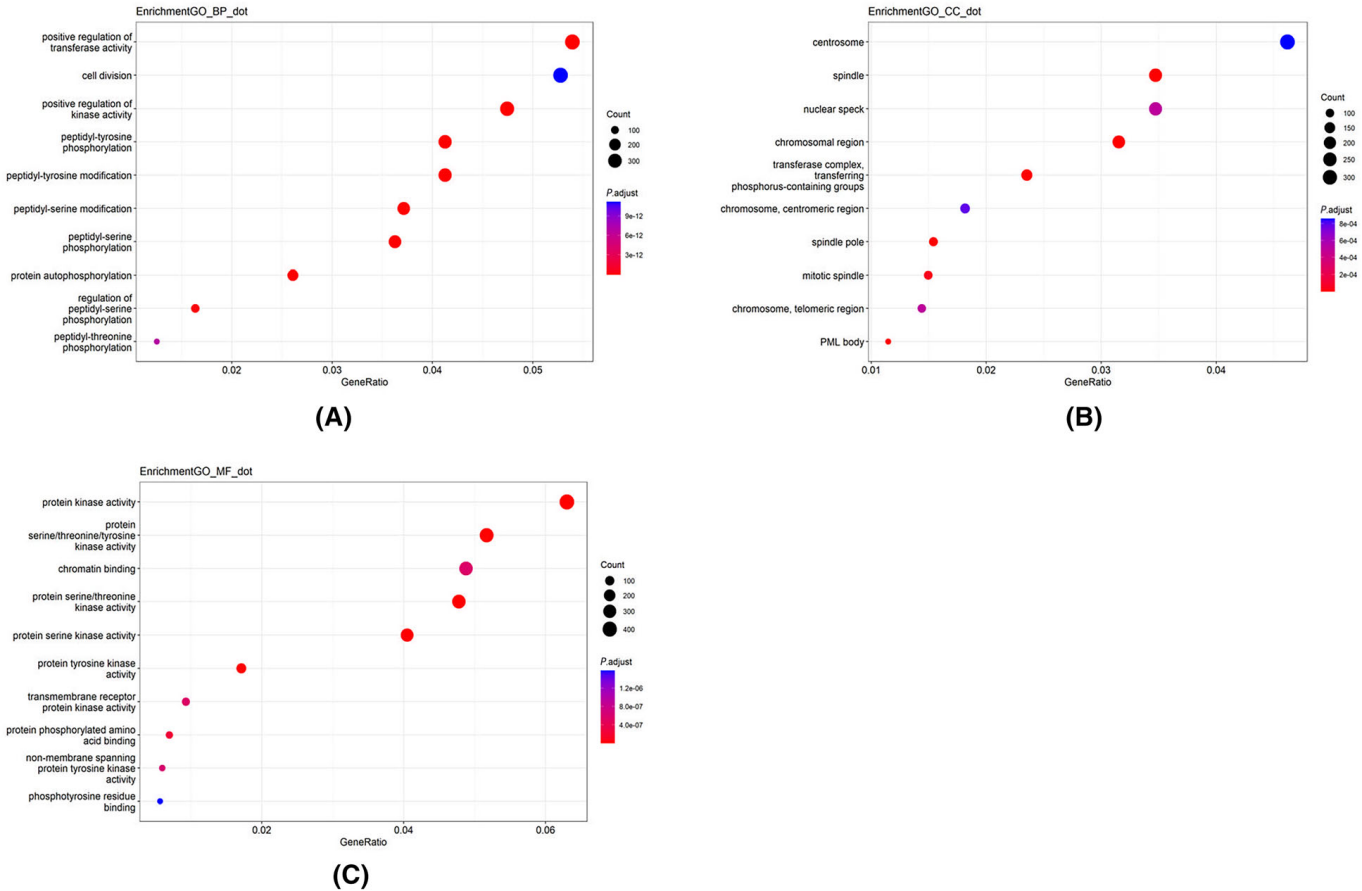
GO function enrichment analysis of proteins predicted to contain changed‐reactivity cysteine. (A) The top 10 most enriched GO terms of biological processes (BP). (B) The top 10 most enriched GO terms of cellular components (CC). (C) The top 10 most enriched GO terms of molecular functions (MF).

**Fig. 6:**
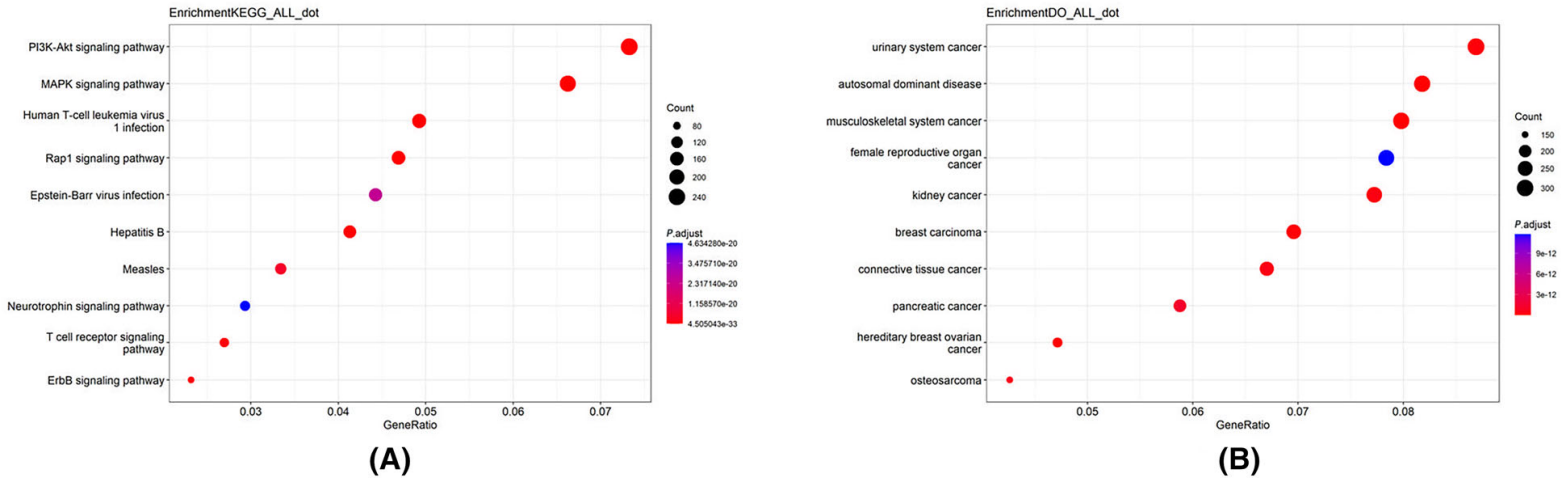
KEGG and DO enrichment analysis of proteins predicted to contain changed‐reactivity cysteine. (A) The top 10 most enriched KEGG terms. (B) The top 10 most enriched DO terms.

Using the final selected tri‐classification predictor, we predicted changes in the reactivity of cysteines on the phosphorylated human proteome. A total of 22 238 phosphorylated proteins from the human genome were obtained from the UniProt database [27]. Subsequently, 207 286 peptides with a length of 21 AAs, centered on cysteine residues, were generated and 11 peptides containing unusual amino acid residues were deleted. The remaining 207 275 peptides were then predicted by our XGBoost model, resulting in the identification of 90 807 sites with changed reactivity, containing 55 273 sites with increased reactivity, and 35 534 sites with decreased reactivity. Using the UniProt database, we mapped the 17 602 proteins predicted to contain changed‐reactivity cysteines to 8440 genes (9206 were not found and 4 were repeated). GO functional enrichment analysis, KEGG pathway analysis, and DO enrichment analysis are conducted on these three gene sets. Figures 5 and 6 show the 10 items with the highest enrichment in each module for the changed set.

Gene Ontology enrichment analysis of proteins with sites of changed reactivity show that these proteins are enriched on chromosomal regions and various cytoskeletons related to cell division, closely associated with the positive regulation of transferase and kinase activity, and phosphorylation of various amino acids. These proteins are localized in a variety of membrane‐to‐nuclear signaling pathways as well as viral infection and several disease pathways. DO enrichment analysis shows these proteins to be significantly associated with autosomal dominant diseases and numerous cancers. Proteins containing only increased or decreased sites have their characteristics, and their enrichment results are shown in Figs [Link], [Link], [Link], [Link]. Our model predicted 5492 proteins only contain sites of increased reactivity, 3583 only contain sites of decreased reactivity, and 8527 contain both.

Using the UniProt database, we mapped the 8527 proteins predicted to contain both decreased‐reactivity and increased‐reactivity cysteines to 4567 genes (3985 not found and 25 repeated). GO functional enrichment analysis, KEGG pathway analysis, and DO enrichment analysis was conducted on these three gene sets. Figures S6 and S7 show the 10 items with the highest enrichment in each module for the changed set.

Similarly, the 3583 proteins predicted with only decreased reactivity were mapped to 2109 genes (1487 not found and 13 repeated) while the 5492 with only increased‐reactivity cysteines were mapped to 1764 genes (3734 not found and 6 repeated). The results of their enrichment analysis are shown in Figs [Link], [Link], [Link], [Link].

Some differences in enrichment can be seen between sites with decreased and increased reactivity. Proteins containing only decreased sites are more associated with mRNA processing and enrichment is relatively significant in autosomal dominant diseases. Proteins containing only increased sites are more associated with phosphorylation processes and protein kinase activity and are significantly enriched in multiple neurodegenerative disease pathways. Overall, it appears that these phosphorylated proteins with changed reactivity are closely related to cell division and signaling, they are found in a variety of disease‐related pathways, particularly cancer, autosomal dominant diseases, and viral infections. The enrichment analysis proves the great clinical value of studying changes in cysteine reactivity in phosphorylated proteins.

In this study, we have developed a machine learning model called XGBoost to predict the reactivities of cysteine influenced by phosphorylation events. Since we did not currently find previous studies that fully aligned with our predicted goals, our algorithm was compared with several common machine learning algorithms, including support vector machine, Gaussian Naive Bayes, Logistic Regression, and random forest. The results indicated that XGBoost outperformed several other machine learning algorithms. Our method has successfully introduced machine learning into the field of cysteine reactivity change prediction with excellent performance, providing a new means of predicting changes in cysteine reactivity, and effectively addressing the time‐consuming and labor‐intensive nature of experimental methods.

While promising results were achieved, there are opportunities for enhancement. First, the consideration of phosphorylation sites currently excludes the tyrosine sites. The inclusion of tyrosine sites might help to build a more comprehensive prediction model. Second, the structural features of the protein account only for the location of the disordered regions but lacking more detailed information on the local structure. Additionally, the relatively small size of the benchmark dataset, particularly the limited number of cysteine sites with increased reactivity, might constrain the learning capacity of the model. To further improve the performance of the proposed model, acquiring more experimentally validated reactivity‐changed cysteine residues for training purposes would be beneficial.

Future directions for our model include the following: collecting more high‐quality experimental data to expand the model's training dataset and improve prediction accuracy; further investigating the biochemistry of cysteine to improve feature engineering and feature selection for the model; and improving the model's interpretability so that researchers can understand how the model makes its predictions and validate their plausibility. Our model can help researchers to better understand the role that cysteine reactivity changes in phosphorylated proteins play in biological processes, with promising applications in biomedical research and precision drug development.

## Conclusion

In this study, we leveraged cysteine reactivity as a valuable indicator of protein function. We introduced machine learning techniques to classify the changes in cysteine reactivity influenced by phosphorylation events into three categories: decreased, unchanged, and increased. A variety of feature encoding methods were used, resulting in a 1468‐dimensional feature set. We adopted XGBoost as the final training algorithm to predict the effect of phosphorylation events on cysteine reactivity.

Our model has shown to be effective, demonstrating that phosphorylation‐dependent changes in cysteine reactivity are predictable. Our results highlighted the critical influence of features such as disordered regions of proteins, proximal sites of S/T phosphorylation, and amino acid composition, on the predicted results. Additionally, the function enrichment analysis has indicated that cysteine with changed reactivity is closely associated with several diseases, including various cancers. This implied the great clinical value of studying changes in the reactivity of cysteines.

## Conflict of interest

The authors declare no conflict of interest.

## Author contributions

JC performed the experiments and wrote the original draft, and YX reviewed and edited the paper.

## Supporting information


**Data S1.** The benchmark dataset.

**Fig. S1.** EN scores with different thresholds. The highest scores and the corresponding thresholds are marked with black vertical lines. (a) EN score for the changed/unchanged samples. (b) EN score for the increased/decreased samples.

**Fig. S2.** IUPRED score distribution.

**Fig. S3.** Distribution of P_dis of changed/unchanged sample. (a) The boxplot of S‐P_dis. (b) The boxplot of S/T‐P_dis.

**Fig. S4.** Average difference of amino acid pair composition between the sample sets. (a) Average difference of changed/unchanged sample. The bar above the abscissa indicates that the changed samples' average value of the composition is higher; otherwise, the unchanged samples' is higher. (b) Average difference of increased/decreased sample. The bar above the abscissa indicates that the increased samples' average value of the composition is higher; otherwise, the decreased samples' is higher.

**Fig. S5.** Performance of classifiers with various fragment lengths. (a) ROC curve of occurrence classifiers. (b) PR curve of occurrence classifiers. (c) ROC curve of direction classifiers. (d) PR curve of direction classifiers. (e) ACC of concatenated classifiers. (f) F1‐score of concatenated classifiers.

**Fig. S6.** GO function enrichment analysis of proteins predicted to contain both decreased‐reactivity and increased‐reactivity cysteine. (a) The top 10 most enriched GO terms of biological processes (BP). (b) The top 10 most enriched GO terms of cellular components (CC). (c) The top 10 most enriched GO terms of molecular functions (MF).

**Fig. S7.** KEGG and DO enrichment analysis of proteins predicted to contain both decreased‐reactivity and increased‐reactivity cysteine. (a) The top 10 most enriched KEGG terms. (b) The top 10 most enriched DO terms.

**Fig. S8.** GO function enrichment analysis of proteins predicted to contain only decreased‐reactivity cysteine. (a) The top 10 most enriched GO terms of biological processes (BP). (b) The top 10 most enriched GO terms of cellular components (CC). (c) The top 10 most enriched GO terms of molecular functions (MF).

**Fig. S9.** KEGG and DO enrichment analysis of proteins predicted to contain only decreased‐reactivity cysteine. (a) The top 10 most enriched KEGG terms. (b) The top 10 most enriched DO terms.

**Fig. S10.** GO function enrichment analysis of proteins predicted to contain only increased‐reactivity cysteine. (a) The top 10 most enriched GO terms of biological processes (BP). (b) The top 10 most enriched GO terms of cellular components (CC). (c) The top 10 most enriched GO terms of molecular functions (MF).

**Fig. S11.** KEGG and DO enrichment analysis of proteins predicted to contain only increased‐reactivity cysteine. (a) The top 10 most enriched KEGG terms. (b) The top 10 most enriched DO terms.

**Table S1.** Most important 10 features in the elastic network. The form “aa_1_aa_2_” indicates amino acid pairs spaced at 0 and the form “aa_1__aa_2_” indicates amino acid pairs spaced at 1.
**Table S2.** Results of baseline occurrence classifiers without feature selection.
**Table S3.** Results of occurrence classifiers after feature selection.
**Table S4.** Results of baseline direction classifiers without SMOTE‐Tomek nor feature selection.
**Table S5.** Results of direction classifiers after SMOTE‐Tomek resampling.
**Table S6.** Results of direction classifiers after SMOTE‐Tomek resampling and feature selection.
**Table S7.** Results of XGBoost with and without SMOTE‐Tomek resampling.
**Table S8.** Results of the direct tri‐classification based on XGBoost.
**Table S9.** Results of occurrence classifiers with various fragment lengths.
**Table S10.** Results of direction classifiers with various fragment lengths.
**Table S11.** Results of concatenated classifiers with various fragment lengths.

## Data Availability

The data that support the findings of this study are available in the Supporting Information of this article.
