# Supplementary figures and images for: Predicting cysteine reactivity changes upon phosphorylation using XGBoost
Source: FEBS Open Bio. 2023 Nov 20;14(1):51–62. doi: 10.1002/2211-5463.13737 (PMC10761938; doi:10.1002/2211-5463.13737)

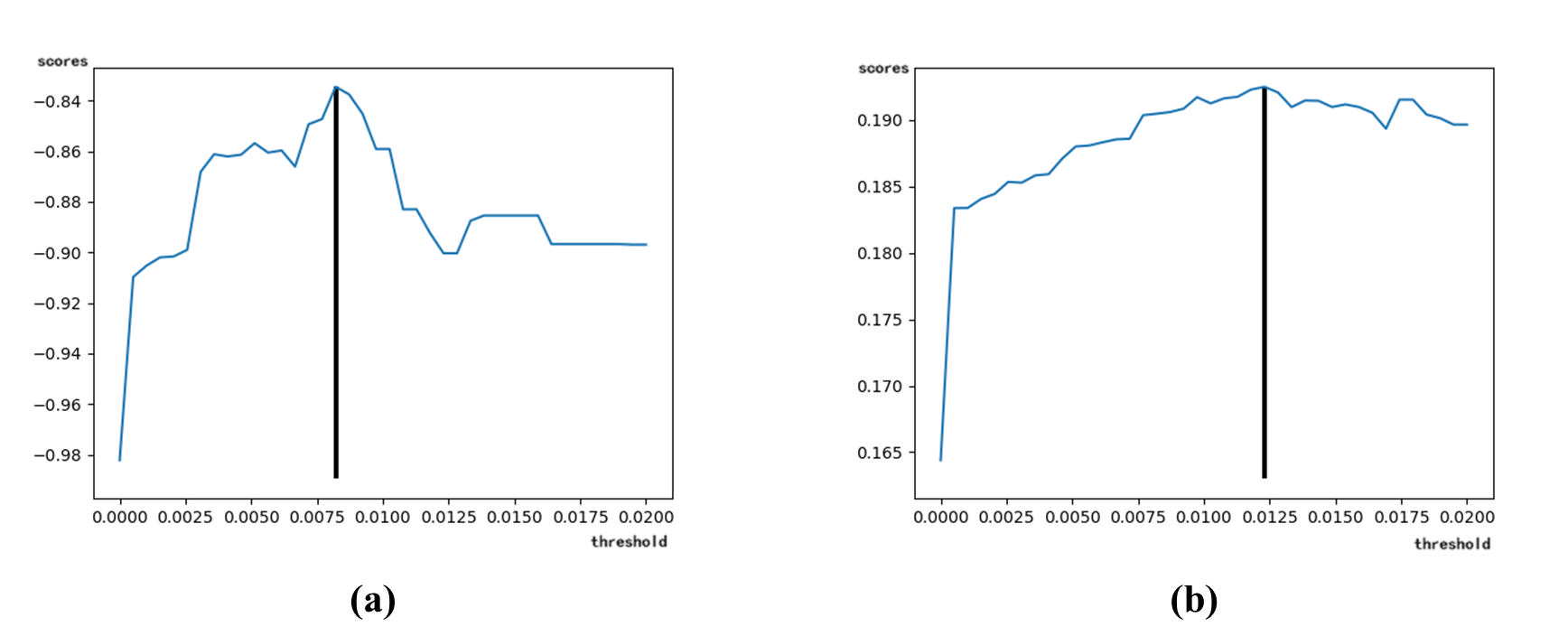

Supplement: Supplementary file 2 — Fig. S1. EN scores with different thresholds. The highest scores and the corresponding thresholds are marked with black vertical lines. (a) EN score for the changed/unchanged samples. (b) EN score for the increased/decreased samples. [file FEB4-14-51-s006.tif]

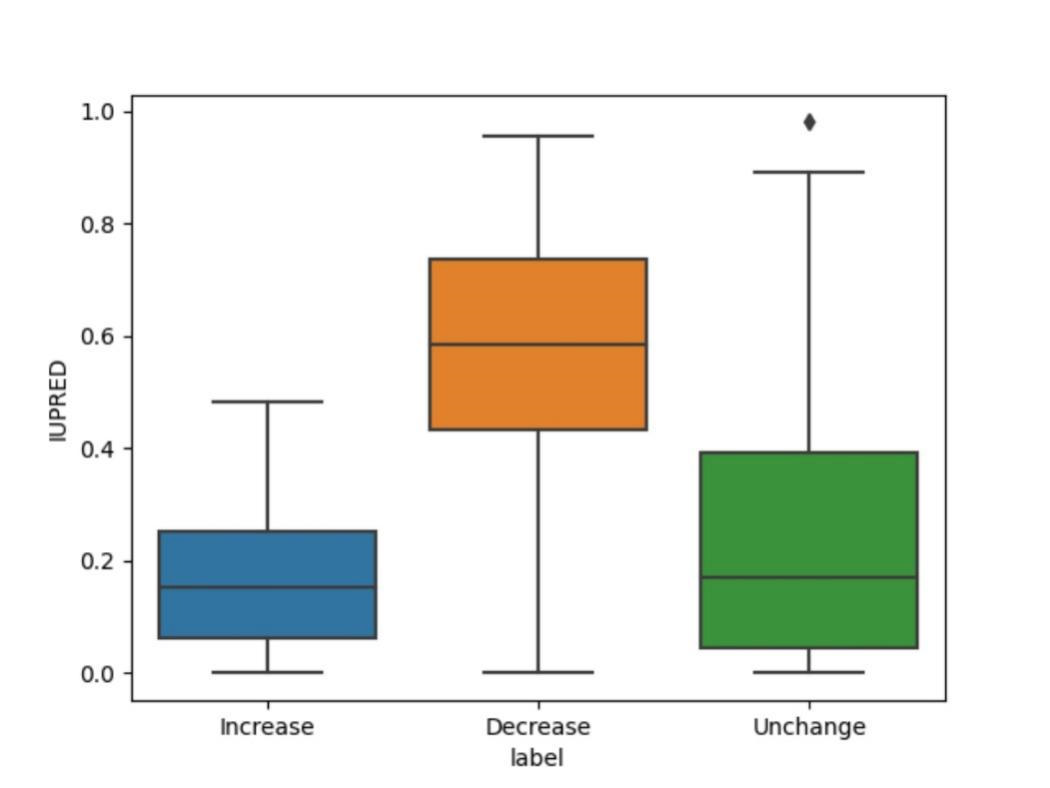

Supplement: Supplementary file 3 — Fig. S2. IUPRED score distribution. [file FEB4-14-51-s012.tif]

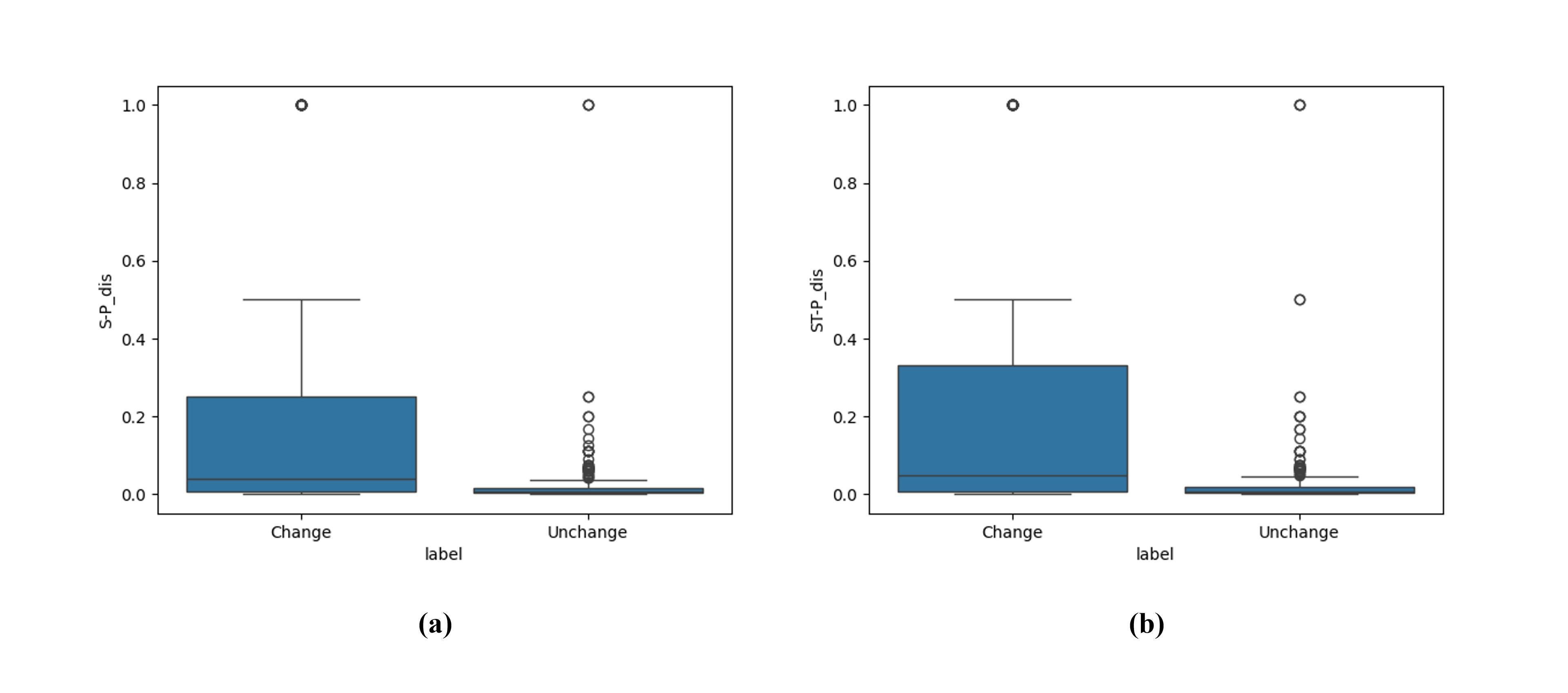

Supplement: Supplementary file 4 — Fig. S3. Distribution of P_dis of changed/unchanged sample. (a) The boxplot of S‐P_dis. (b) The boxplot of S/T‐P_dis. [file FEB4-14-51-s005.tif]

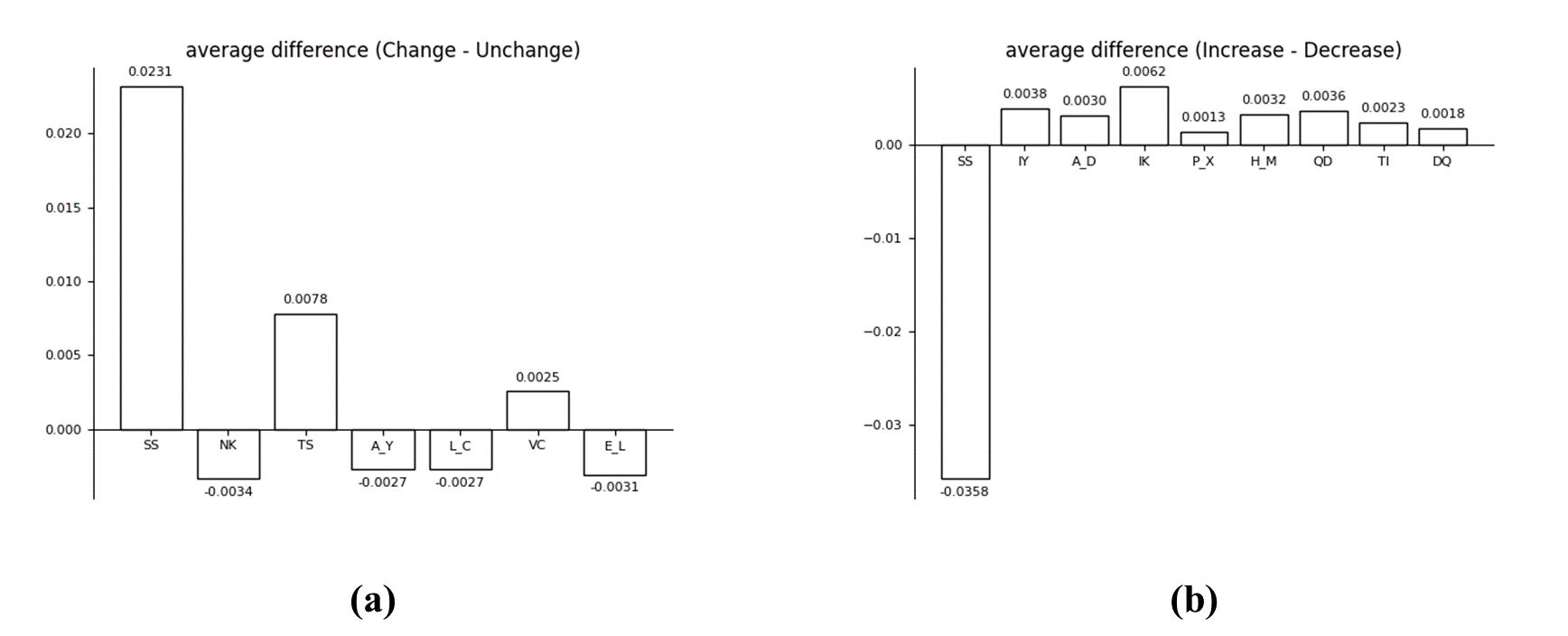

Supplement: Supplementary file 5 — Fig. S4. Average difference of amino acid pair composition between the sample sets. (a) Average difference of changed/unchanged sample. The bar above the abscissa indicates that the changed samples' average value of the composition is higher; otherwise, the unchanged samples' is higher. (b) Average difference of increased/decreased sample. The bar above the abscissa indicates that the increased samples' average value of the composition is higher; otherwise, the decreased samples' is higher. [file FEB4-14-51-s011.tif]

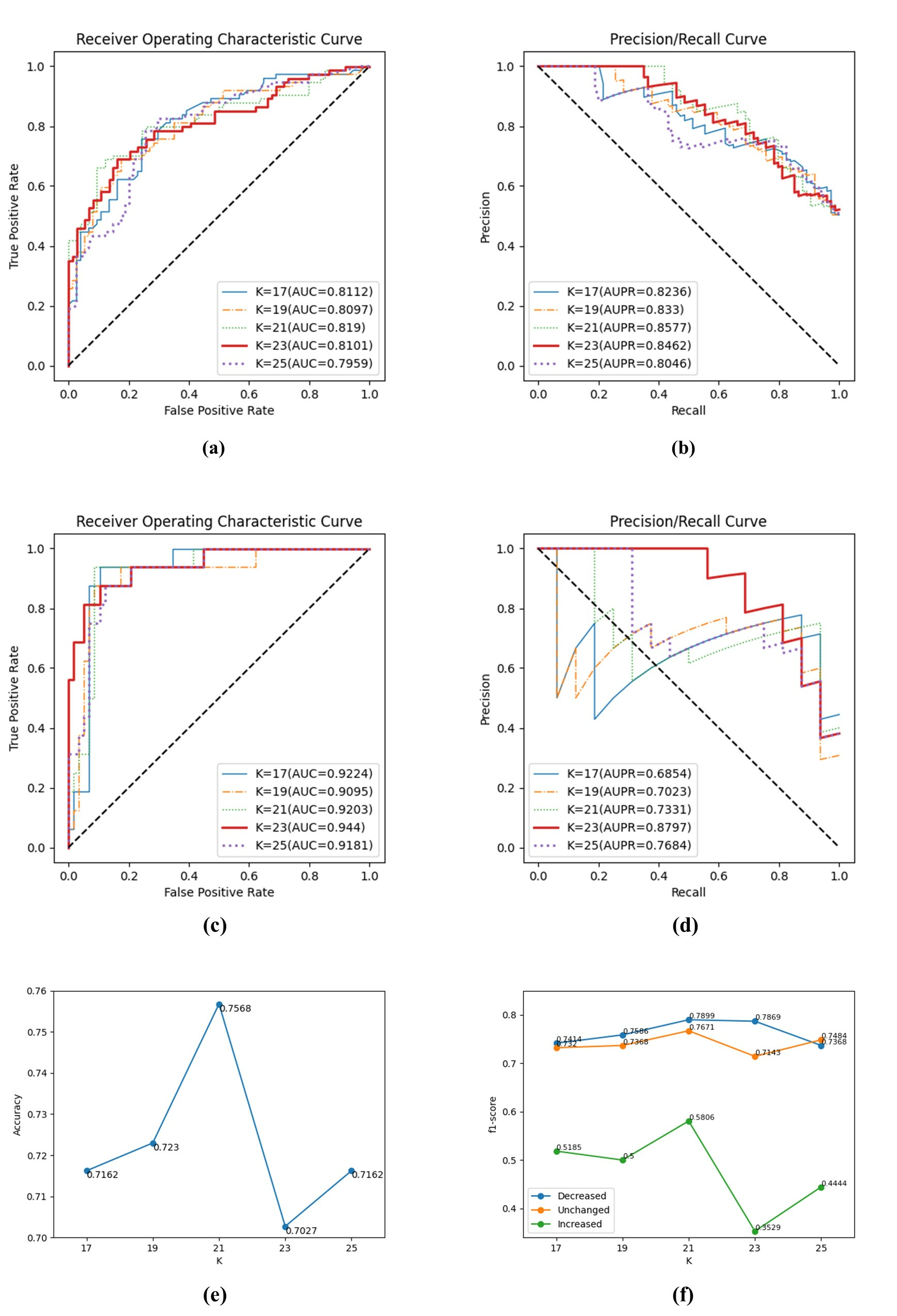

Supplement: Supplementary file 6 — Fig. S5. Performance of classifiers with various fragment lengths. (a) ROC curve of occurrence classifiers. (b) PR curve of occurrence classifiers. (c) ROC curve of direction classifiers. (d) PR curve of direction classifiers. (e) ACC of concatenated classifiers. (f) F1‐score of concatenated classifiers. [file FEB4-14-51-s004.tif]

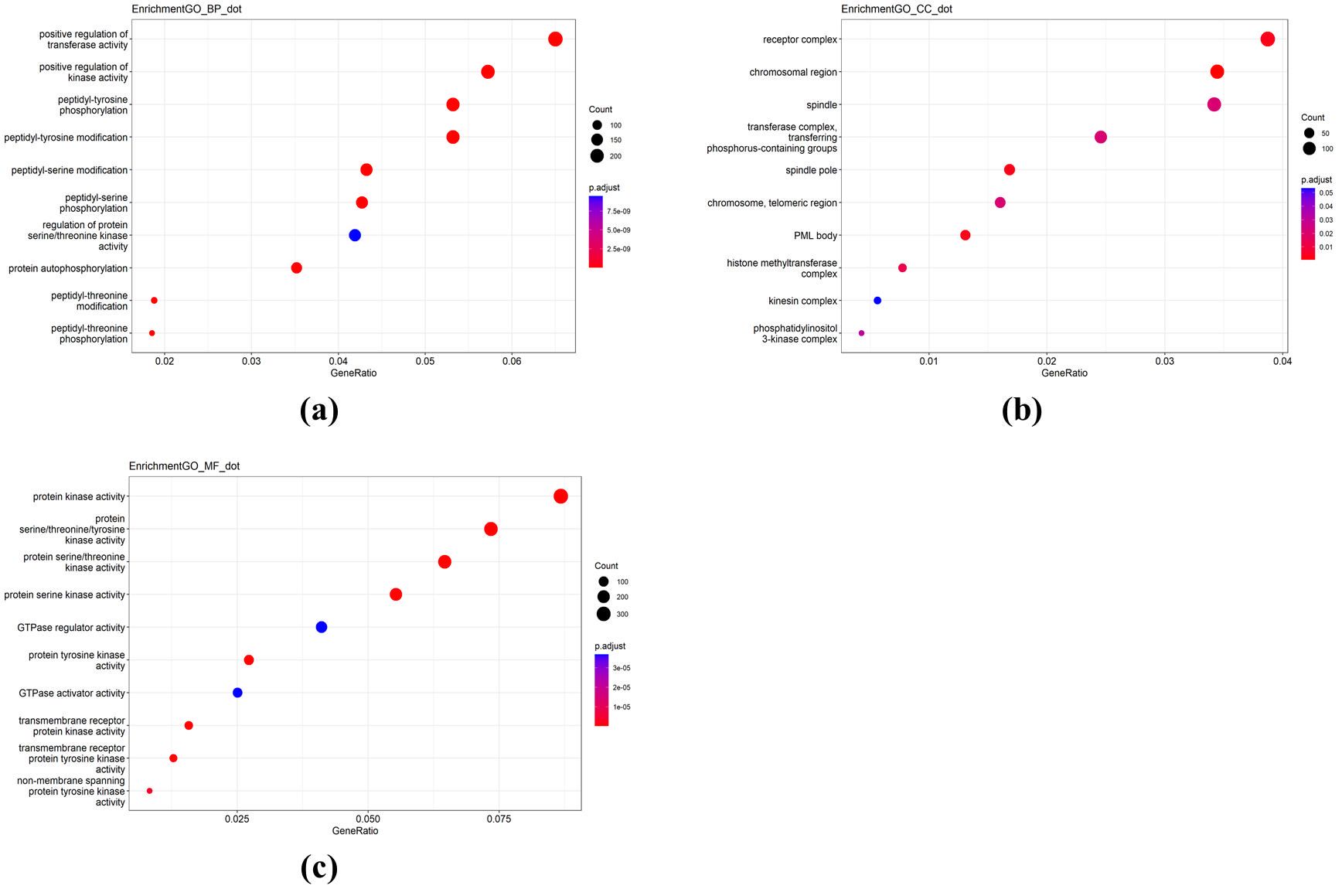

Supplement: Supplementary file 7 — Fig. S6. GO function enrichment analysis of proteins predicted to contain both decreased‐reactivity and increased‐reactivity cysteine. (a) The top 10 most enriched GO terms of biological processes (BP). (b) The top 10 most enriched GO terms of cellular components (CC). (c) The top 10 most enriched GO terms of molecular functions (MF). [file FEB4-14-51-s009.tif]

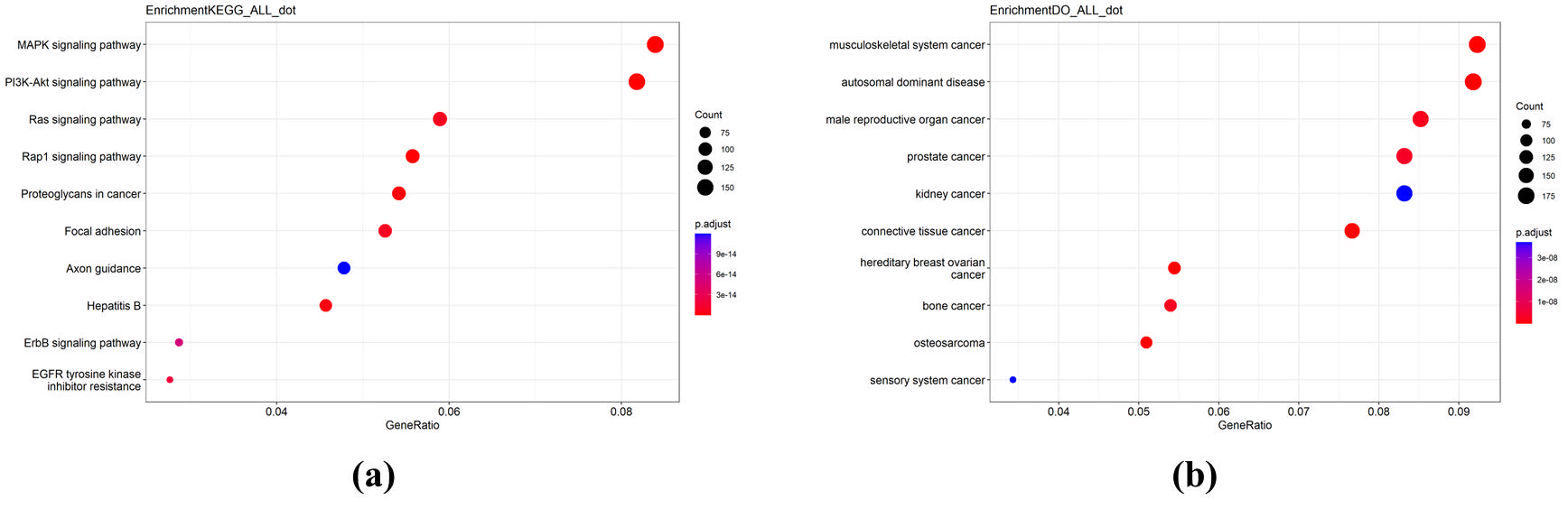

Supplement: Supplementary file 8 — Fig. S7. KEGG and DO enrichment analysis of proteins predicted to contain both decreased‐reactivity and increased‐reactivity cysteine. (a) The top 10 most enriched KEGG terms. (b) The top 10 most enriched DO terms. [file FEB4-14-51-s002.tif]

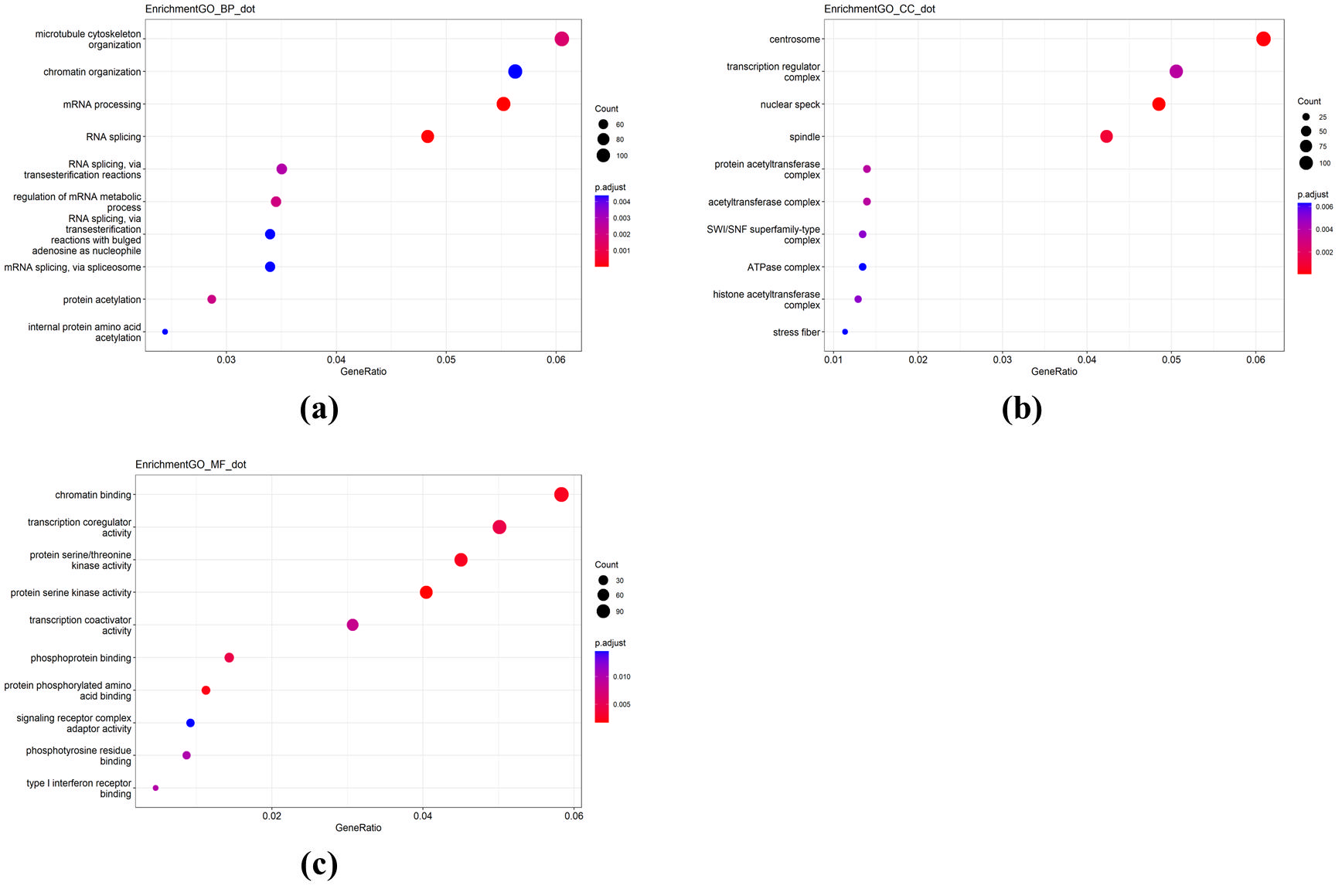

Supplement: Supplementary file 9 — Fig. S8. GO function enrichment analysis of proteins predicted to contain only decreased‐reactivity cysteine. (a) The top 10 most enriched GO terms of biological processes (BP). (b) The top 10 most enriched GO terms of cellular components (CC). (c) The top 10 most enriched GO terms of molecular functions (MF). [file FEB4-14-51-s007.tif]

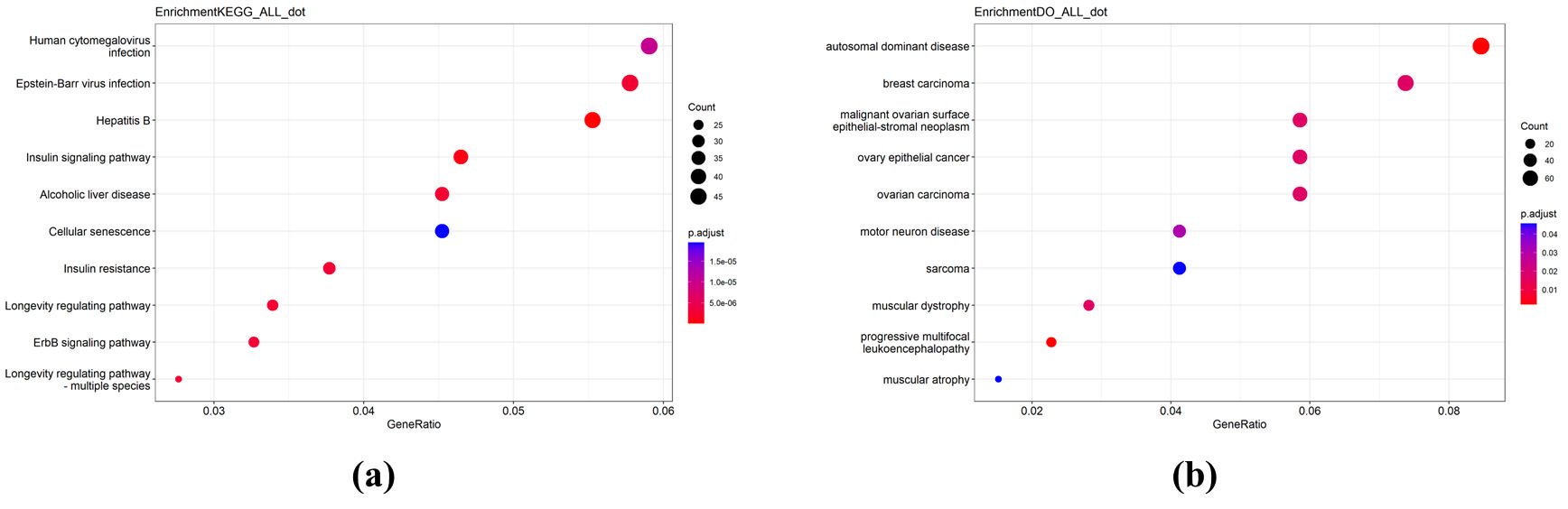

Supplement: Supplementary file 10 — Fig. S9. KEGG and DO enrichment analysis of proteins predicted to contain only decreased‐reactivity cysteine. (a) The top 10 most enriched KEGG terms. (b) The top 10 most enriched DO terms. [file FEB4-14-51-s013.tif]

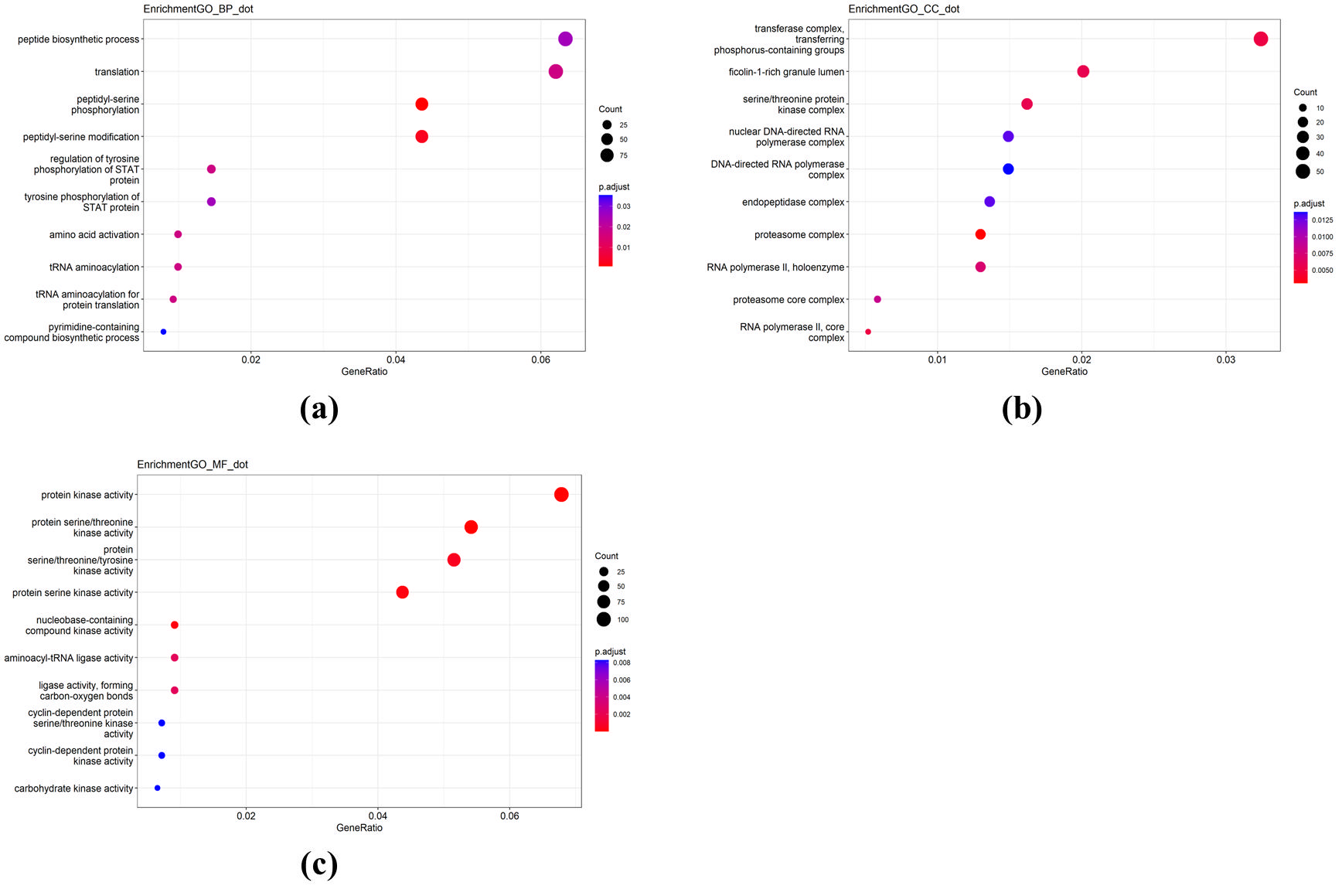

Supplement: Supplementary file 11 — Fig. S10. GO function enrichment analysis of proteins predicted to contain only increased‐reactivity cysteine. (a) The top 10 most enriched GO terms of biological processes (BP). (b) The top 10 most enriched GO terms of cellular components (CC). (c) The top 10 most enriched GO terms of molecular functions (MF). [file FEB4-14-51-s008.tif]

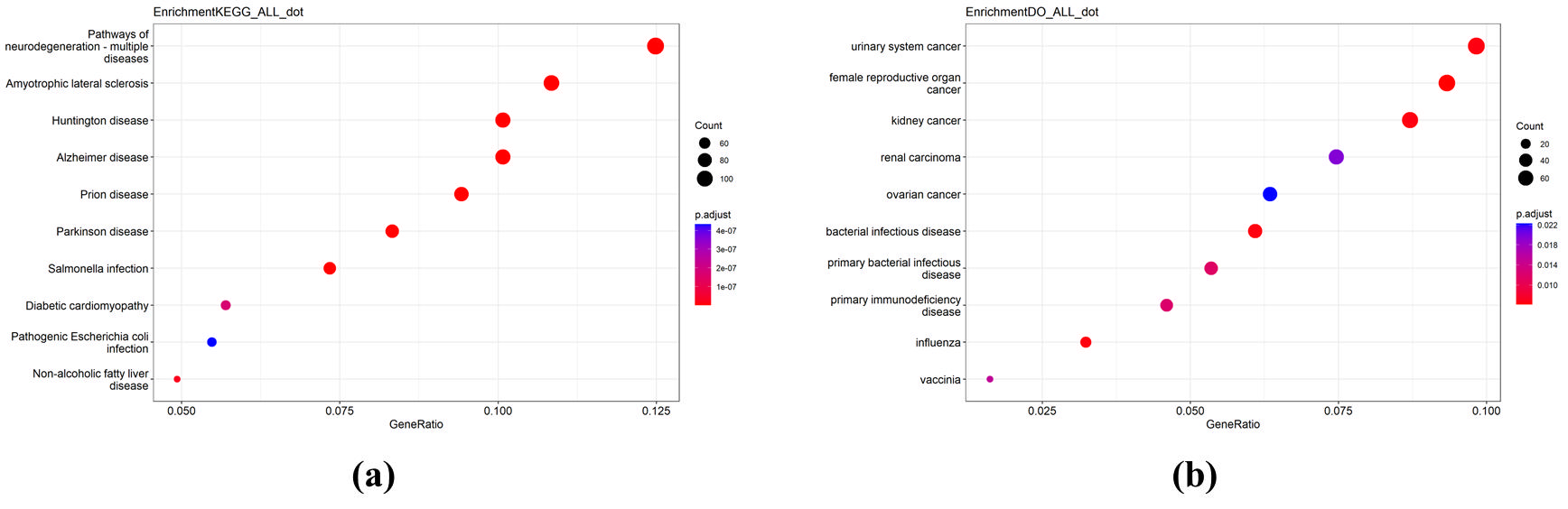

Supplement: Supplementary file 12 — Fig. S11. KEGG and DO enrichment analysis of proteins predicted to contain only increased‐reactivity cysteine. (a) The top 10 most enriched KEGG terms. (b) The top 10 most enriched DO terms. [file FEB4-14-51-s001.tif]
