# Supplementary material for: Predicting cysteine reactivity changes upon phosphorylation using XGBoost
Source: FEBS Open Bio. 2023 Nov 20;14(1):51–62. doi: 10.1002/2211-5463.13737 (PMC10761938; doi:10.1002/2211-5463.13737)
Supplement: Supplementary file 13 — Table S1. Most important 10 features in the elastic network. The form “aa1aa2” indicates amino acid pairs spaced at 0 and the form “aa1_aa2” indicates amino acid pairs spaced at 1. Table S2. Results of baseline occurrence classifiers without feature selection. Table S3. Results of occurrence classifiers after feature selection. Table S4. Results of baseline direction classifiers without SMOTE‐Tomek nor feature selection. Table S5. Results of direction classifiers after SMOTE‐Tomek resampling. Table S6. Results of direction classifiers after SMOTE‐Tomek resampling and feature selection. Table S7. Results of XGBoost with and without SMOTE‐Tomek resampling. Table S8. Results of the direct tri‐classification based on XGBoost. Table S9. Results of occurrence classifiers with various fragment lengths. Table S10. Results of direction classifiers with various fragment lengths. Table S11. Results of concatenated classifiers with various fragment lengths. [file FEB4-14-51-s010.docx]

**Table S1.** **The most important 10 features in the elastic network.** The form 'aa_1_aa_2_' indicates amino acid pairs spaced at 0 and the form 'aa_1__aa_2_' indicates amino acid pairs spaced at 1.

| Reactivity | Features |
| --- | --- |
| occurrence | IUPRED, ‘SS', S-P_dis, S/T-P_dis, 'NK', 'TS', 'A_Y', 'L_C', 'VC', 'E_L' |
| direction | IUPRED, 'SS', 'IY', 'A_D', 'IK', 'P_X', 'H_M', 'QD', 'TI', 'DQ' |

Table S2. Results of baseline occurrence classifiers without feature selection.

|  | ACC | Sn | Sp | Precision | F1-score | MCC | AUC | AUPR |
| --- | --- | --- | --- | --- | --- | --- | --- | --- |
| SVM | 0.7027 | 0.5946 | 0.8108 | 0.7586 | 0.6667 | 0.4152 | 0.7715 | 0.8118 |
| NB | 0.5743 | 0.8649 | 0.2838 | 0.5470 | 0.6702 | 0.1826 | 0.5766 | 0.5463 |
| LR | 0.5811 | 0.6216 | 0.5405 | 0.5750 | 0.5974 | 0.1627 | 0.6393 | 0.6584 |
| RF | 0.7365 | 0.6486 | 0.8243 | 0.7869 | 0.7111 | 0.4804 | 0.8062 | 0.8358 |
| XGB | 0.7365 | 0.7027 | 0.7703 | 0.7536 | 0.7273 | 0.4741 | 0.8218 | 0.8551 |

Table S3. Results of occurrence classifiers after feature selection.

|  | ACC | Sn | Sp | Precision | F1-score | MCC | AUC | AUPR |
| --- | --- | --- | --- | --- | --- | --- | --- | --- |
| SVM | 0.6824 | 0.6216 | 0.7432 | 0.7077 | 0.6619 | 0.3676 | 0.7590 | 0.8197 |
| NB | 0.7027 | 0.6351 | 0.7703 | 0.7344 | 0.6812 | 0.4091 | 0.7285 | 0.7102 |
| LR | 0.7500 | 0.6622 | 0.8378 | 0.8033 | 0.7259 | 0.5079 | 0.7703 | 0.8112 |
| RF | 0.7365 | 0.7162 | 0.7568 | 0.7465 | 0.7310 | 0.4734 | 0.7973 | 0.8298 |
| XGB | 0.7703 | 0.7838 | 0.7568 | 0.7632 | 0.7733 | 0.5407 | 0.8190 | 0.8577 |

Table S4. Results of baseline direction classifiers without SMOTE-Tomek nor feature selection.

|  | ACC | Sn | Sp | Precision | F1-score | MCC | AUC | AUPR |
| --- | --- | --- | --- | --- | --- | --- | --- | --- |
| SVM | 0.7973 | 0.0625 | 1.0000 | 1.0000 | 0.1176 | 0.2228 | 0.8287 | 0.6205 |
| NB | 0.6486 | 0.2500 | 0.7586 | 0.2222 | 0.2353 | 0.0083 | 0.5043 | 0.2177 |
| LR | 0.8378 | 0.4375 | 0.9483 | 0.7000 | 0.5385 | 0.4645 | 0.8728 | 0.6678 |
| RF | 0.8108 | 0.1250 | 1.0000 | 1.0000 | 0.2222 | 0.3173 | 0.8966 | 0.7755 |
| XGB | 0.8784 | 0.8125 | 0.8966 | 0.6842 | 0.7429 | 0.6682 | 0.9041 | 0.6763 |

Table S5. Results of direction classifiers after SMOTE-Tomek resampling.

|  | ACC | Sn | Sp | Precision | F1-score | MCC | AUC | AUPR |
| --- | --- | --- | --- | --- | --- | --- | --- | --- |
| SVM | 0.7973 | 0.0625 | 1.0000 | 1.0000 | 0.1176 | 0.2228 | 0.8761 | 0.6366 |
| NB | 0.6351 | 0.1250 | 0.7759 | 0.1333 | 0.1290 | -0.1015 | 0.4504 | 0.2059 |
| LR | 0.8784 | 0.6250 | 0.9483 | 0.7692 | 0.6897 | 0.6202 | 0.8815 | 0.6881 |
| RF | 0.8514 | 0.5000 | 0.9483 | 0.7273 | 0.5926 | 0.5187 | 0.9122 | 0.7443 |
| XGB | 0.8784 | 0.8125 | 0.8966 | 0.6842 | 0.7429 | 0.6682 | 0.9170 | 0.6875 |

Table S6. Results of direction classifiers after SMOTE-Tomek resampling and feature selection.

|  | ACC | Sn | Sp | Precision | F1-score | MCC | AUC | AUPR |
| --- | --- | --- | --- | --- | --- | --- | --- | --- |
| SVM | 0.8108 | 0.2500 | 0.9655 | 0.6667 | 0.3636 | 0.3250 | 0.8438 | 0.5757 |
| NB | 0.5946 | 0.3125 | 0.6724 | 0.2083 | 0.2500 | -0.0133 | 0.4914 | 0.2268 |
| LR | 0.7973 | 0.4375 | 0.8966 | 0.5385 | 0.4828 | 0.3614 | 0.8394 | 0.5165 |
| RF | 0.8649 | 0.6875 | 0.9138 | 0.6875 | 0.6875 | 0.6013 | 0.9240 | 0.7081 |
| XGB | 0.9189 | 0.9375 | 0.9138 | 0.7500 | 0.8333 | 0.7891 | 0.9203 | 0.7331 |

Table S7. Results of XGBoost with and without SMOTE-Tomek resampling.

|  | ACC | Sn | Sp | Precision | F1-score | MCC | AUC | AUPR |
| --- | --- | --- | --- | --- | --- | --- | --- | --- |
| Resample | 0.8784 | 0.8125 | 0.9655 | 0.8966 | 0.7429 | 0.6682 | 0.9143 | 0.8097 |
| Without  resample | 0.9189 | 0.9375 | 0.9138 | 0.7500 | 0.8333 | 0.7891 | 0.9203 | 0.7331 |

Table S8. Results of the direct tri-classification based on XGBoost.

| Conditions | Category | ACC | Precision | Recall | F1-score |
| --- | --- | --- | --- | --- | --- |
| No resampling  No selection | Decreased | 0.7095 | 0.8000 | 0.6897 | 0.7407 |
|  | Unchanged |  | 0.6739 | 0.8378 | 0.7470 |
|  | Increased |  | 0.5000 | 0.1875 | 0.2727 |
| Resampling  No selection | Decreased | 0.7365 | 0.8036 | 0.7759 | 0.7895 |
|  | Unchanged |  | 0.7176 | 0.8243 | 0.7673 |
|  | Increased |  | 0.4286 | 0.1875 | 0.2609 |
| Resampling  Selection | Decreased | 0.6757 | 0.7736 | 0.7069 | 0.7387 |
|  | Unchanged |  | 0.6552 | 0.7703 | 0.7081 |
|  | Increased |  | 0.2500 | 0.1250 | 0.1667 |

## Analysis of fragment length

**Table S9. Results of occurrence classifiers with various fragment lengths.**

| K | ACC | Sn | Sp | Precision | F1-score | MCC | AUC | AUPR |
| --- | --- | --- | --- | --- | --- | --- | --- | --- |
| 17 | 0.7230 | 0.6892 | 0.7568 | 0.7391 | 0.7133 | 0.4470 | 0.8112 | 0.8236 |
| 19 | 0.7297 | 0.7027 | 0.7568 | 0.7429 | 0.7222 | 0.4601 | 0.8097 | 0.8330 |
| 21 | 0.7703 | 0.7838 | 0.7568 | 0.7632 | 0.7733 | 0.5407 | 0.8190 | 0.8577 |
| 23 | 0.7297 | 0.7838 | 0.6757 | 0.7073 | 0.7436 | 0.4622 | 0.8101 | 0.8462 |
| 25 | 0.7365 | 0.6892 | 0.7838 | 0.7612 | 0.7234 | 0.4751 | 0.7959 | 0.8046 |

**Table S10. Results of direction classifiers with various fragment lengths.**

| K | ACC | Sn | Sp | Precision | F1-score | MCC | AUC | AUPR |
| --- | --- | --- | --- | --- | --- | --- | --- | --- |
| 17 | 0.9054 | 0.8750 | 0.9138 | 0.7368 | 0.8000 | 0.7433 | 0.9224 | 0.6854 |
| 19 | 0.9054 | 0.8750 | 0.9138 | 0.7368 | 0.8000 | 0.7433 | 0.9095 | 0.7023 |
| 21 | 0.9189 | 0.9375 | 0.9138 | 0.7500 | 0.8333 | 0.7891 | 0.9203 | 0.7331 |
| 23 | 0.9054 | 0.8125 | 0.9310 | 0.9440 | 0.7879 | 0.7276 | 0.9439 | 0.8797 |
| 25 | 0.8649 | 0.8125 | 0.8793 | 0.6500 | 0.7222 | 0.6413 | 0.9181 | 0.7684 |

**Table S11. Results of concatenated classifiers with various fragment lengths.**

| K | ACC | Precision | | | Recall | | | F1-score | | |
| --- | --- | --- | --- | --- | --- | --- | --- | --- | --- | --- |
|  |  | Decreased | Unchanged | Increased | Decreased | Unchanged | Increased | Decreased | Unchanged | Increased |
| 17 | 0.7162 | 0.7414 | 0.7089 | 0.6364 | 0.7414 | 0.7568 | 0.4375 | 0.7414 | 0.7320 | 0.5185 |
| 19 | 0.7230 | 0.7586 | 0.7179 | 0.5833 | 0.7586 | 0.7568 | 0.4375 | 0.7586 | 0.7368 | 0.5000 |
| 21 | 0.7568 | 0.7705 | 0.7778 | 0.6000 | 0.8103 | 0.7568 | 0.5625 | 0.7899 | 0.7671 | 0.5806 |
| 23 | 0.7027 | 0.7500 | 0.7576 | 0.3333 | 0.8276 | 0.6757 | 0.3750 | 0.7869 | 0.7143 | 0.3529 |
| 25 | 0.7162 | 0.7500 | 0.7160 | 0.5455 | 0.7241 | 0.7838 | 0.3750 | 0.7368 | 0.7484 | 0.4444 |
